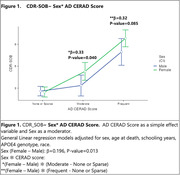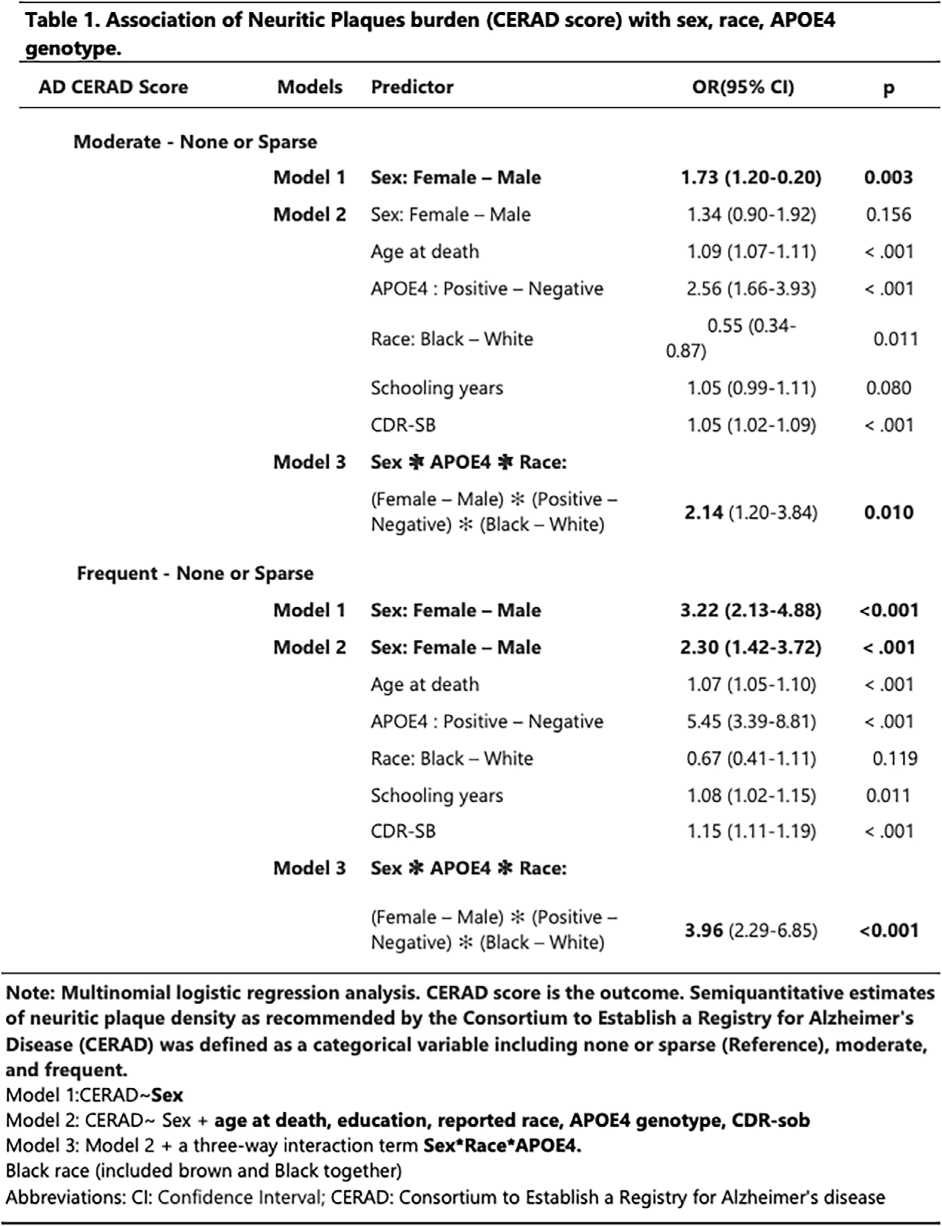# Exploring the Impact of Race, African Ancestry, and APOE4 on Sex Differences in Amyloid Pathology and Cognitive Outcomes in an Admixed Population‐Based Neuropathological Sample

**DOI:** 10.1002/alz.093140

**Published:** 2025-01-03

**Authors:** Maison Abu Raya, Vitor Ribeiro Paes, Renata Elaine Paraizo Leite, Carlos Augusto Pasqualucci, Wilson Jacob‐Filho, Ricardo Nitrini, Claudia Kimie Suemoto, Lea T. Grinberg

**Affiliations:** ^1^ University of california San Francisco, San Francisco, CA USA; ^2^ Global Brain Health institute‐ UCSF, San Francisco, CA USA; ^3^ University of São Paulo Medical School, São Paulo, São Paulo Brazil; ^4^ Physiopathology in Aging Laboratory (LIM‐22), University of Sao Paulo Medical School, São Paulo, São Paulo Brazil; ^5^ Biobank for Aging Studies of the University of São Paulo Medical School, São Paulo Brazil; ^6^ Physiopathology in Aging Laboratory (LIM‐22), University of São Paulo Medical School, São Paulo, São Paulo Brazil; ^7^ Brazilian Brain Bank of the Aging Brain Study Group; University of São Paulo, São Paulo Brazil; ^8^ Biobank for aging studies of the University of São Paulo, São Paulo Brazil; ^9^ Faculdade de Medicina da Universidade de São Paulo, São Paulo Brazil; ^10^ Physiopathology in Aging Laboratory (LIM‐22), University of São Paulo Medical School, São Paulo Brazil; ^11^ Cognitive and Behavioral Neurology Unit ‐ University of São Paulo, São Paulo Brazil; ^12^ Biobank for aging studies of the University of São Paulo Medical School, São Paulo Brazil; ^13^ Physiopathology in Aging Laboratory (LIM‐22), Department of Internal Medicine, University of Sao Paulo Medical School, São Paulo, São Paulo Brazil; ^14^ Division of Geriatrics, Department of Internal Medicine, University of São Paulo Medical School, São Paulo, São Paulo Brazil; ^15^ Memory & Aging Center, Department of Neurology, University of California in San Francisco, San Francisco, CA USA

## Abstract

**Background:**

Prior research investigating sex and racial differences in amyloid pathology burden has yielded inconsistent findings. We examined the impact of sex and other confounding factors on neuritic plaque burden and cognitive outcomes.

**Method:**

This study included 1,857 individuals, with post‐mortem brain tissues, from the Biobank for Aging Studies of the University of São Paulo Medical School, collected from 2004‐2023. In a subset of subjects, global ancestry was analyzed dichotomously, using a 2% cutoff for African Ancestry (AFR), first quartile of the AFR distribution. We employed multinomial logistic regression and general linear models to explore the associations of sex, reported race (White versus Black and Brown groups combined), AFR, and APOE4 genotype on CERAD scores and cognitive outcomes measured by Clinical Dementia Rating Scale Sum of Boxes(CDR‐SOB) while adjusting for age at death and education.

**Result:**

Women (48% of the sample) were older than men (76.8±12.3 yo versus 71.4±12.3 yo, p<0.001). Men had higher educational attainment with a mean(SD) of 5.5(4.2) years and 3.9(3.9) years in women, (p<0.001). Dementia prevalence was higher in women (31% vs.18.5%, p<0.001). The sample was predominantly white (64.7%), followed by black (23.1%), and Asian (2.2%). 76.7% of men and 76.5% of women had AFR+ (p = 0.96). Women had higher odds of ‘Frequent’ CERAD scores with OR(_95%_CI) = 2.3(1.42‐3.72), p<0.001) **Table 1 (Frequent‐Sparse or None) model 2**. Black race was associated with lower odds of ‘Moderate’ CERAD scores compared to whites. A significant three‐way interaction among sex, APOE4, and race showed that Black men without APOE4 had lower odds of ‘Moderate’ and ‘Frequent’ CERAD scores **(Table 1)**. Similar trends were found with Sex*APOE4*AFR. Testing the association of CERAD scores with cognitive outcomes, higher CERAD scores were associated with lower CDR‐SOB (P<0.001), and sex moderated this effect showing that women with ‘Moderate’ CERAD scores had worse cognitive outcomes than men **(Figure 1)**. Notably, black race **(Figure 2),** but not AFR **(Figure 3),** modified the interaction between sex and CERAD on cognitive outcomes.

**Conclusion:**

Women exhibited increased susceptibility to amyloid pathology, resulting in more pronounced cognitive impairment, suggesting that sex‐specific genetic, biological, and sociocultural factors may play a role in women’s higher AD vulnerability.